# Application of Biobased Solvents in Asymmetric Catalysis

**DOI:** 10.3390/molecules27196701

**Published:** 2022-10-08

**Authors:** Margherita Miele, Veronica Pillari, Vittorio Pace, Andrés R. Alcántara, Gonzalo de Gonzalo

**Affiliations:** 1 Department of Chemistry, University of Torino, Via Giuria 7, 10125 Torino, Italy; 2 Department of Pharmaceutical Sciences, University of Vienna, Josef-Holaubek Platz 2, 1090 Vienna, Austria; 3 Department of Chemistry in Pharmaceutical Sciences, Faculty of Pharmacy, Complutense University of Madrid, Plaza de Ramón y Cajal s/n, 28040 Madrid, Spain; 4 Department of Organic Chemistry, University of Seville, c/ Profesor García González 1, 41014 Seville, Spain

**Keywords:** biobased solvents, green chemistry, biocatalysis, organocatalysis, metal catalysis

## Abstract

The necessity of more sustainable conditions that follow the twelve principles of Green Chemistry have pushed researchers to the development of novel reagents, catalysts and solvents for greener asymmetric methodologies. Solvents are in general a fundamental part for developing organic processes, as well as for the separation and purification of the reaction products. By this reason, in the last years, the application of the so-called green solvents has emerged as a useful alternative to the classical organic solvents. These solvents must present some properties, such as a low vapor pressure and toxicity, high boiling point and biodegradability, and must be obtained from renewable sources. In the present revision, the recent application of these biobased solvents in the synthesis of optically active compounds employing different catalytic methodologies, including biocatalysis, organocatalysis and metal catalysis, will be analyzed to provide a novel tool for carrying out more ecofriendly organic processes.

## 1. Introduction

The development of methods for the incorporation of stereogenic centers into a molecule to enhance its three-dimensional structure, therefore conferring superior properties, represents an ever-present challenge in Synthetic and Medicinal Chemistry. In fact, the ability to produce stereoisomeric compounds is of great importance in drug discovery due to the different biological activities of the possible enantiomers of a molecule and the consistently high demand of drugs with a finer efficacy and limited side effects [1]. This prompted the investigation of new chemistry and the development of innovative tools and solutions in asymmetric synthesis [2]. In fact, stereoselective reactions permit the installation of a new stereogenic unit (chiral center, chiral axis or chiral plane) giving access to the unequal proportions of possible chiral stereoisomers, making unequal the energies of activation for the diastereomeric transition states allowing the formation of an enantiomeric excess higher than 99% [3].

Any component able to induce dissymmetric influence on the reaction can be taken into account; in this sense, normally, the asymmetry is created by the catalyst, although the solvent effect has to be also considered. In fact, solvents do not only represent just the medium in which the reaction is carried out; it has to be considered that they account for the largest proportion of chemicals used in a pharmaceutical process, so that solvents represent the major waste in these processes [4]. Therefore, as the development of sustainable and cost-effective chemical processes has become one of the top objectives of chemists nowadays, there is an intense research focus and attention on the replacement of hazardous solvents with less dangerous ones, as well as to their recovery and reuse [5,6,7,8,9,10,11,12,13,14,15,16,17,18], aligned with the twelve Principles of Green Chemistry, established some years ago by Anastas and Warner [19]. As most of the organic reactions are carried out in solutions, solvents are maybe one of the most active areas in the search of green processes [20]. The proper selection of a solvent is a key step when developing chemical processes for the preparation of high-added-value compounds. Apart from its physicochemical properties and ecotoxicity, the solvent employed in asymmetric reactions should also present an easy recovery and purification as well as an easy affordability. In view of these requirements, the “classical” organic solvents present several drawbacks. In fact, they are usually obtained from non-renewable sources and in general they have a high toxicity and flammability, thus presenting a high environmental impact as well as generating wastes that must be removed. By these reasons, different alternatives to this classical approach are required when applying catalytic procedures. Water can be established as the first choice for a “green solvent” search, as it is an environmentally friendly solvent, but its application in catalytic organic syntheses is hampered by some drawbacks: (a) water presents a high polarity, leading to solubility issues of the organic substrates which usually lead to low concentrations; (b) in addition, water presents an intrinsic reactivity, resulting in undesired side reactions. In the last years, neoteric solvents (both ionic liquids and deep eutectic solvents) have appeared as a valuable alternative for developing sustainable catalytic processes. Ionic liquids (ILs) are salts liquids at temperatures lower than 100 °C, which present low vapor pressure and flammability and high thermal stability [21,22]. However, ILs present some environmental and toxicity issues which has slowed down their establishment as a green alternative to classical solvents. Deep eutectic solvents (DESs), compounds formed by the combination of a hydrogen-bond donor with a hydrogen-bond acceptor, have appeared as greener alternative to ILs, with several examples of their application in catalytic procedures [23,24,25]. Other alternatives that can be considered for carrying out reactions in green solvents are the use of supercritical solvents (mainly supercritical CO_2_) [26,27]. A recent approach to the “classical” organic solvents in asymmetric chemical synthesis has been the use of the biomass-derived solvents, the so-called biosolvents [28,29]. These solvents, which are very similar to the “classical” solvents, present a set of properties, such as low toxicity, high biodegradability and being obtained from renewable sources, which convert them into a valuable alternative for the development of sustainable chemical processes. 

Nowadays several compounds that are obtained from renewable sources and present a low ecotoxicity can be applied as green solvents with several applications. Among all of them, in the present review, we will focus on those biobased solvents that have demonstrated their utility as reaction media in asymmetric processes. 

## 2. Properties of the Typical Biobased Solvents Employed in Asymmetric Catalysis

Although the use of water is extensively investigated [30], several compounds obtained from renewable sources, presenting a low ecotoxicity, can be applied as green solvents in chemical processes [30]. Glycerol and its derivatives; mixtures of carbohydrates; secondary metabolites of plants, such as limonene or α-pinene; fatty acid methyl esters (biodiesel); esters of lactic or gluconic acid; biobased ethers; and dihydrolevoglucosenone and γ-valerolactone are considered as biobased solvents [28,29]. Among all of them, in the present review, we will focus on those solvents that have demonstrated their utility as reaction media in catalytic asymmetric procedures leading to chiral products, such as the ethereal solvents 2-methyltetrahydrofuran (2-MeTHF) and cyclopentyl methyl ether (CPME) and the heterocyclic cycloalakanone dihydrolevoglucosenone or Cyrene^TM^ (Table 1 shows some of these three solvents’ properties). Unconventional ethereal solvents have appeared as a valuable tool [31]. Thus, 2-MeTHF, available through the catalytic reduction of furfural and levulinic acid [32], is emerging as a very promising alternative [33,34,35]. Compared to THF, 2-MeTHF shows a lower water miscibility (140 g/L [36]), higher stability and lower volatility (2-MeTHF has melting and boiling points of −136 and 80 °C, whereas for THF these are −108 and 66 °C, respectively). 2-MeTHF displays a low toxicity and neither mutagenicity nor genotoxicity characteristics [37]. Supporting the safe use of 2-MeTHF in the pharmaceutical industry, a No-Observed-Adverse-Effect Level (NOAEL) of 250 mg/kg day has been reported [38]. Even though 2-MeTHF is generally considered to be readily degradable [34], there are not much data illustrating the degrading pathways. Anyhow, regardless of its biogenic origin, 2-MeTHF is still problematic due to its high flammability [39], even considering that its flash point (−11 °C) is higher than that of hexane (−30 °C) [40]. Similarly, the industrially produced solvents through a 100% atom-economical reaction, such as cyclopentyl methyl ether (CPME), proved to be an evaluable “green” alternative in a variety of organic reactions and we could anticipate also in asymmetric tactics [41,42,43]. CPME is characterized by a high boiling point (106 °C) and a low freezing point (−140 °C). The better thermal stability of these solvents permits the employment within a wide range of temperatures, and in the case of CPME, the low vaporization energy enables an easy recovery and reuse via classical distillation methods [43]. Both are characterized by a partial miscibility with water, therefore permitting clean and easy work-up procedures and a drastic decrease in classical organic solvents for extracting the reaction products [34]. Taking into account the toxicological point of view, CPME and 2-MeTHF are characterized by a lower acute or subchronic toxicity and, according to the toxicological assays, a negative genotoxicity and mutagenicity during the exposure. Unlike 2-methyltetrahydrofuran (2-MeTHF), CPME shows a particularly high resistance to PO formation [41]. If for THF and 2-MeTHF the formation of peroxides could not be avoided, and thus the use of stabilizers is required, CPME is characterized by a major stability toward autoxidation under an oxygen atmosphere [44], as highlighted in the development of rechargeable Li–air batteries [45]. According to these ecofriendly properties, CPME can be considered a greener alternative, together with 2- MeTHF, to more problematic ethereal solvents, such as diethyl ether (Et_2_O), tetrahydrofuran (THF), 1,2-dimethoxyethane (DME) [43], 1,4-dioxane and methyl *tert*-butyl ether (MTBE) [46].

A very promising new biosolvent is dihydrolevoglucosenone or Cyrene^TM^ (registered by Merck), which can be obtained from cellulose through a pyrolysis and/or hydrogenation process [47,48]. Dihydrolevoglucosenone presents a high boiling point (226 °C [13]), is only poorly ecotoxic (OECD No. 201, 202 and 209) and has no mutagenicity (OECD No. 471 and 487), with an LD50 > 2000 mg/kg (OECD No. 423, acute toxicity method) [49]. Comparing the Kamlet–Abboud–Taft and Hansen solubility parameter, Clark et al. found that dihydrolevoglucosenone owns: (a) the dispersion parameters closest to DMSO (18.8 vs. 18.4 MPa), (b) the polarity closest to dimethylacetamide (DMAc, 10.6 vs. 11.5 MPa) and (c) its hydrogen-bonding-like interactions were most similar to *N*-methyl-2-pyrrolidone (NMP, 6.9 vs. 7.2 MPa) [50]. This solvent can be derivatized with 1,2-diols, allowing the formation of novel solvent types. Cyrene has shown some recent applications as a solvent in catalysis, which will be mentioned in the next sections [51]. 

## 3. Biobased Solvents in Asymmetric Metal-Based Catalysis 

Notably, a big portion of asymmetric synthesis includes chiral organometallic reagents, such as enantiomerically enriched organolithiums and Grignard reagents [52]. Organometallic reagents appear to show a better performance in solvents featuring ether functionalities due to their Lewis-base behavior, able to disaggregate organometallic species modulating their reactivity [53]. Unfortunately, the most employed solvents, such as THF, tend to react with highly basic carbanions, precluding the use of high temperatures in order to avoid undesired reactions, such as the α-cleavage of THF in the presence of lithium (e.g., the half-life in the presence of *n*-BuLi 10 min at 35 °C) [54]. The most serious drawback of ethereal solvents is the formation of peroxides (POs) under an oxygen atmosphere, limiting storage and handling safety, but also possible irritation, genotoxicity and mutagenicity during the exposure [55].

**Figure 1:**
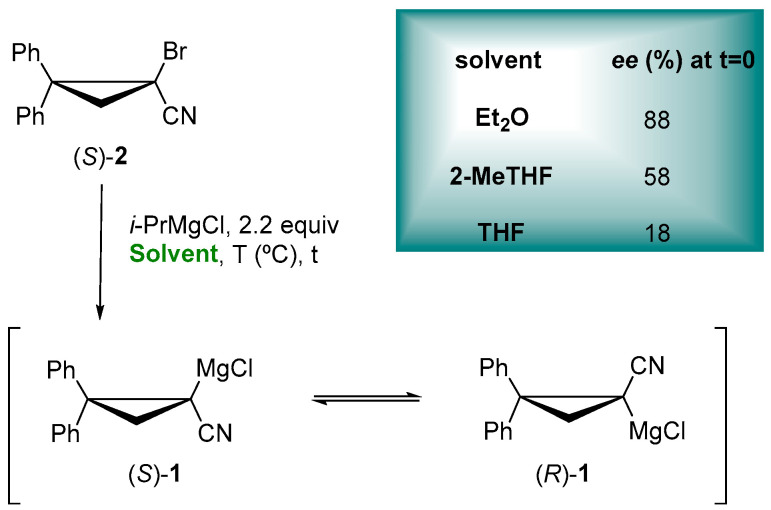
Enantioenriched α-cyanocyclopropyl Grignard reagents.

Ethereal solvents can dramatically affect the configurational stability of enantioenriched organometallic reagents, as showed for the enantioselective trapping of configurationally stable lithiated [56,57] and magnesiated nitriles [58], thus having an impact on the key characteristic for the synthetic utility of them in asymmetric synthesis. In the preparation of the enantioenriched cyclopropylnitrile Grignard reagent (*S*)-**1,** through the Mg/Br exchange of bromonitrile (*S*)-**2** with *i*-PrMgCl, it exhibits a more configurational retention in Et_2_O < 2-MeTHF < THF (Figure 1), proving the role of ethereal solvent not only for the configurational stability but also for the stereochemical fidelity of the Mg/Br exchange, in accordance with Gawley and coworkers, as reported for the racemization rates of a lithiated Boc-pyrrolidine [59]. In fact, the coordinating ability of 2-MeTHF is intermediate between Et_2_O and THF, so that the rate of enantiomerization is faster than with Et_2_O but slower than with THF [60].

**Figure 2:**
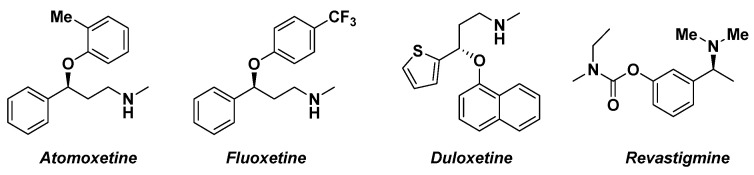
Pharmacologically active homochiral analogues.

The asymmetric synthesis of enantioenriched alcohols is representing a pivotal step in the synthesis of pharmaceutical molecules (Figure 2) [61]. The commonly employed methodologies involve the use of biocatalysts [62], transition metals [63], chiral hydrides [64] or homogenous asymmetric reduction based on oxazaborolidine [65].

**Figure 3:**
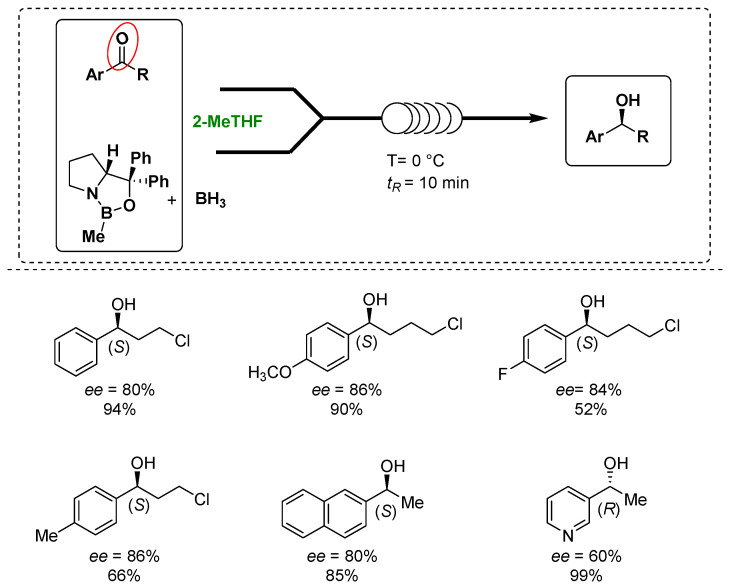
Luisi’s asymmetric reduction of ketones to alcohols under microfluidic conditions in 2-MeTHF.

Luisi’s group exploited the use of the Corey–Bakshi–Shibata (CBS) oxazaborolidine as a catalyst to control the enantioselectivity of the reduction of prochiral arylketones under homogeneous conditions in combination with the use of chip microreactors and 2-MeTHF as a greener solvent without employing any additives to develop a more sustainable process, as shown in Figure 3 [66]. In fact, the microreactors and continuous flow technologies have attracted the interest of the pharmaceutical and chemical industry due to the lower costs, improved reliability, safety and sustainability and the continuous processes, demonstrating, together with the solvent choice, their importance in the design of greener processes [67]. During the optimization phase, the screening of the solvents presented 2-MeTHF to provide a slightly better enantiomeric excess of the desired product in comparison with THF. Under the optimized reaction conditions, the targeted enantiopure alcohols were obtained in up to a 99% yield and 82% enantiomeric excess in only 10 min. In addition, the use of flow technology and the in-line monitoring and work up procedures helped to reduce the amount of the catalyst and optimize the process also in term of sustainability.

**Figure 4:**
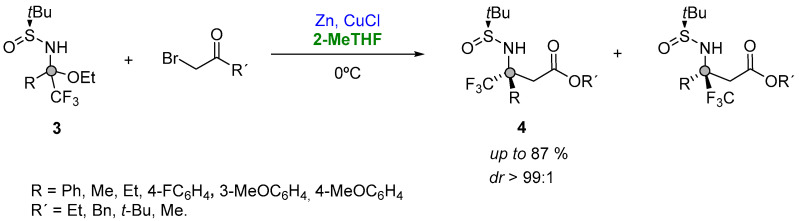
Organozinc addition to a quaternary carbon center.

Starting from a series of aromatic and aliphatic trifluoromethylketones enroute to bench-stable trifluoromethyl *N*-tert-butanesulfinyl ketoimines (Figure 4, (**3**)), and subsequently reacting with the Reformatsky reagent formed in situ, Grellepois described the asymmetric synthesis of β-alkyl(aryl) β-trifluoromethyl β-amino acids (**4**) containing a quaternary stereocenter at the β position (Figure 4). The reaction was explored at room temperature in a variety of solvents (DME, Et_2_O, THF, 2-Me-THF, CH_2_Cl_2_, DMF or acetonitrile), showing a major stereoselectivity for the addition in 2-MeTHF. In addition, the use of this solvent was found beneficial in order to further decrease the temperature at 0 °C, thus having a better diastereoselectivity (88:12 instead of 86:14) and yield [68].

**Figure 5:**
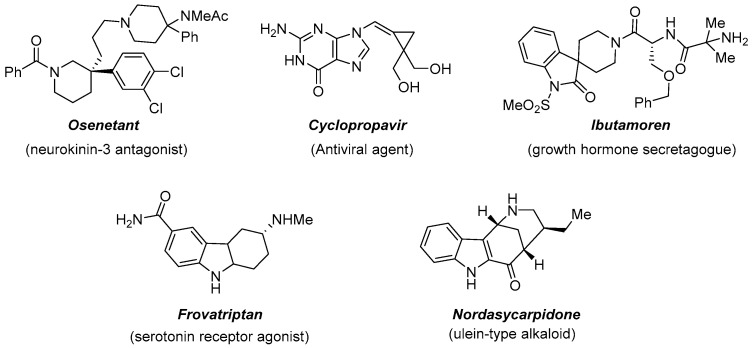
Biologically active substances featuring a quaternary stereocenter.

The introduction of a three-dimensional characteristic into a molecule, through the installation of a quaternary stereocenter, is representing an important tool to access various molecular scaffolds useful in drug discovery and design [2]. The evidence is, for example, the presence of nitrogen heterocycles in natural products or as useful building blocks in organic synthesis (Figure 5).

Among asymmetric synthesis procedures, a particular advantage is represented by the use of transition metal catalysts, able to tune the interaction of a variety of ligands forming a number of possible diastereomeric combinations, and the interaction between the chiral metal complex and the achiral substrate represents the diastereotopic interaction leading to the asymmetric process [69]. In this context, Dyson and Jessop pointed out the importance of the interactions between a solvent, catalyst, substrates and products influencing both the speed and the selectivity of reactions [70]. Thus, solvents may tune the selectivity and efficiency of a given process and, in particular recent applications, show CPME [71,72] and 2-MeTHF [73,74] as useful green solvents in asymmetric catalysis. 

**Figure 6:**
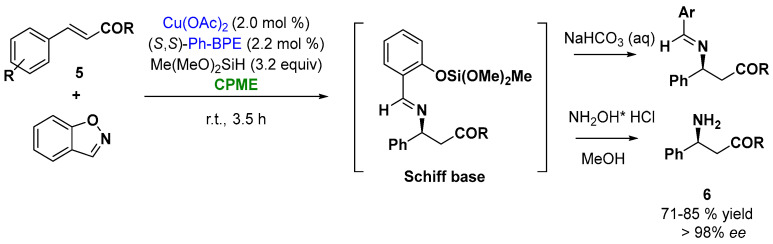
Enantioselective Cu-catalyzed hydroamination of cinnamoyl derivatives.

Among all the examples in the enantioselective catalysis by metals, the ability of copper to coordinate a broad range of chiral ligands makes copper catalysis of particular interest [75]. Thus, the ability of copper to react with an electrophilic aminating reagent, such as 1,2-benzisoxazole, allowed the synthesis of enantioenriched β-amino acid derivatives, important building blocks for the synthesis of natural products and small molecule pharmaceuticals. Buchwald and coworkers reported an enantioselective CuH-catalyzed hydroamination protocol of α, β-unsaturated carbonyl compounds (cinnamate derivatives and *tert*-butyl cinnamate derivatives, Figure 6, (**5**)), with commercially available 1,2-benzisoxazole, in the presence of the ligand (*S*,*S*)-Ph-BPE. The reaction involves the in situ silylation of the carboxylic acid, the hydroamination to give the Schiff base, which upon hydrolysis releases the β-amino acid derivatives (**6**). The screening of the solvents indicated that CPME was an excellent solvent providing high yields and enantioselectivities (higher than 96% *ee*), as depicted in Figure 6 [76].

**Figure 7:**
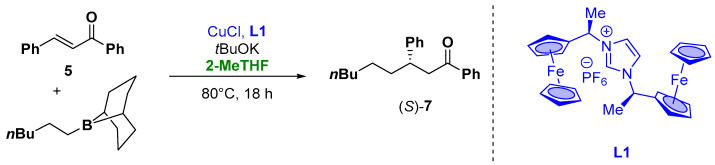
Enantioselective Michael-type addition of organoboranes to chalcones.

Moreover, organometallic reagents are suitable for the enantioselective Cu-catalyzed conjugate addition to α,β-unsaturated carbonyl compounds [77,78,79,80]. Capable for this transformation, alkyl organoboranes or alkenyl organoboranes with the use of ferrocene-carbene ligands have also been presented. The catalytic activity of the ferrocene-carbene ligand **L1** (Figure 7) was examined for the enantioselective addition of alkylborane to α,β-unsaturated ketones, using 15 mol% of CuCl with 15 mol% of chiral ligand in the presence of 30 mol% of *t*BuOK as a base. The target compound **7** was isolated in a 20% yield due to the decomposition of the ligand to vinylferrocene during the reaction and with 34% *ee* (Figure 7). To improve the reaction conditions from an environmental and health point of view, the “greener”, less hygroscopic and higher boiling solvent 2-MeTHF was employed. In fact, the use of THF, due to the high reaction temperature and time, resulted in the complete evaporation of the solvent. These prompt the use of different aromatic and etheric solvents (such as dioxane or toluene) which lead to the zero conversion into the product. On the other hand, the use of 2-MeTHF, due to the higher boiling point and its lower affinity to the water, resulted in the more efficient and suitable choice also in the case of the prolonged heating of the reaction mixture [81].

**Figure 8:**
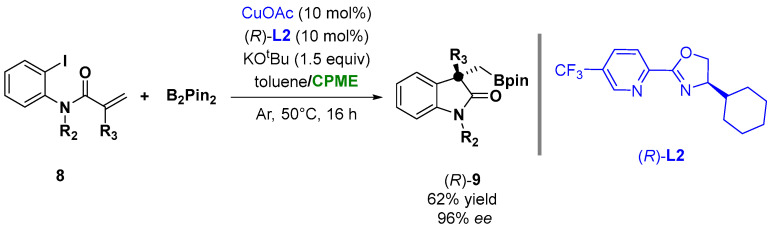
Asymmetric α-borylmethyl oxindoles from *o*-iodoanilides.

The coordination of copper with the chiral ligand **L2** (Figure 8) was also successfully employed in the case of copper-catalyzed asymmetric arylboronation of *N*-(*o*-iodoaryl) acrylamides (**8**) with bis(pinacolato)diboron (B_2_Pin_2_), leading to chiral oxindoles bearing BPin-containing all-carbon quaternary centers (**9**), as shown in Figure 8. The screening of the solvent indicated that a mixture of toluene/CPME was the optimal choice to achieve 96% *ee* and 62% yield [82].

**Figure 9:**
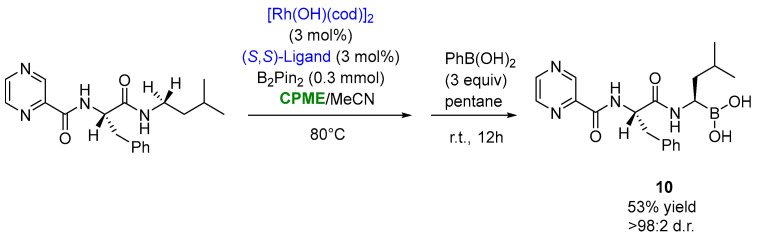
Synthesis of bortezomib through asymmetric Rh-catalyzed borylation of aa leucine analogue.

The use of CPME appeared to also be productive, in a mixture 1:1 with MeCN, in the optimized synthesis of bortezomib ((**10**), Figure 9), an anti-cancer drug consisting of three structural units linked together by peptide bonds: pyrazinoyl, L-phenylalanyl and L-boroleucinyl. This compound was obtained via the stereoselective rhodium-catalyzed borylation of the N-adjacent C−H bond of the pro-boroleucine residue with bis(pinacolato)diboron (pinB-Bpin) in the presence of the triisopropylsilyloxy (TIPS)-modified BINOL-based mono-phosphite ligand at 80 °C for 36 h, furnishing bortezomib in a 53% yield and >98:2 d.r., after the transesterification of the pinacol ester with phenylboronic acid (Figure 9) [83].

**Figure 10:**
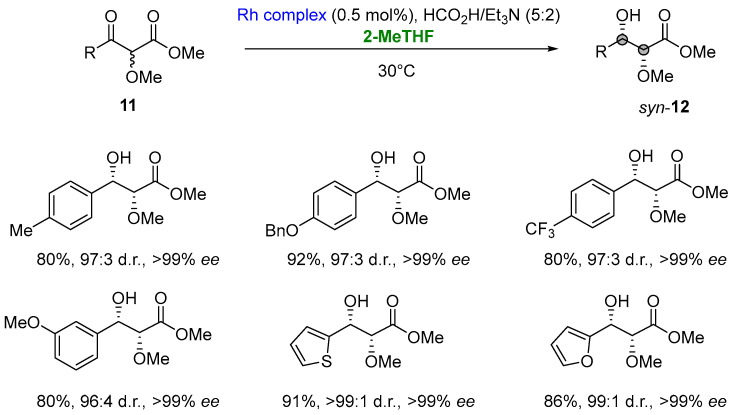
Combined reductive-DKR en route to *syn*-α-alkoxy β-hydroxyesters.

Notably, in the asymmetric transfer hydrogenation (ATH) of α-alkoxy β-ketoesters (**11**) via dynamic kinetic resolution (DKR) involving a rhodium complex (*N*-pentafluorophenylsulfonyl-DPEN-based tethered Rh(III) complex, 2-MeTHF was selected as a greener solvent to develop an environmentally sustainable procedure [19,84]. ATH/DKR access the desired enantiomerically enriched *syn*-α-alkoxy β-hydroxyesters (**12**) in high yields (68–97%), high levels of diastereocontrol (95:5 to 99:1 d.r.) and excellent enantioselectivities (>99% *ee*) (Figure 10) [85].

**Figure 11:**
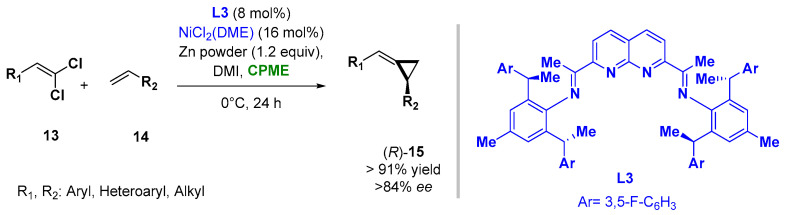
Asymmetric Ni-catalyzed alkylidene-cyclopropanes from olefines and *gem*-dichloroalkenes.

The use of CPME and 2-MeTHF was also reported when carrying out asymmetric Nickel-catalyzed procedures as asymmetric alkylidenecyclopropanations and [2 + 2 + 2] cycloadditions to en route substituted pyridines, respectively. In the reductive Ni-catalyzed enantioselective alkylidene transfer from 1,1-dichloroalkenes (**13**) to olefins (**14**), the use of CPME as a solvent, in combination with the additive 1,3-dimethyl-2-imidazolidinone (DMI) and ligand **L3**, allowed the improving of the yields and enantioselectivity of the process at 0 °C, giving access to a broad range of alkylidenecyclopropanes (**15**), as indicated in Figure 11 [86].

**Figure 12:**
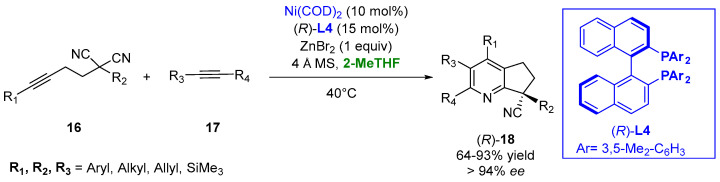
Chiral pyridine derivatives from functionalized malononitriles and alkynes.

The use of the bio-renewable 2-MeTHF provides a better yield and enantioselectivity in the case of a nickel-catalyzed asymmetric [2 + 2 + 2] cycloaddition in the presence of ligand (*R*)-**L4** of the disubstituted malononitriles (**16**) and alkynes (**17**) to generate all-carbon quaternary center-containing substituted pyridines (**18**) under mild conditions. The use of zinc halide as an additive was crucial for the success of the process, and the better outcome of the reaction in 2-MeTHF appeared to be reasonable due to the weak coordination ability of this solvent to the transition metals and the high solubility of the zinc halides (Figure 12) [87]. 

## 4. Biosolvents in Asymmetric Organocatalysis

The use of (relatively) small organic molecules as catalysts in organic reactions has experienced overwhelming interest in the last few years. The Nobel Prize in Chemistry in 2021 has confirmed the importance of this catalytic approach for the preparation of chiral compounds [88,89,90,91]. Organocatalytic methods offer some advantages over other catalytic approaches, such as the operational simplicity of the organocatalytic processes (normally tolerant to water and air), the stability and non-toxicity of the catalysts, the tolerance to different functional groups, the high diversity of the organic molecules available and the possibility of being modified, thus obtaining a wide set of compounds to be employed as organocatalysts in these reactions. Several approaches have also been performed to develop greener organocatalytic methodologies, thus including the application of green solvents.

**Figure 13:**
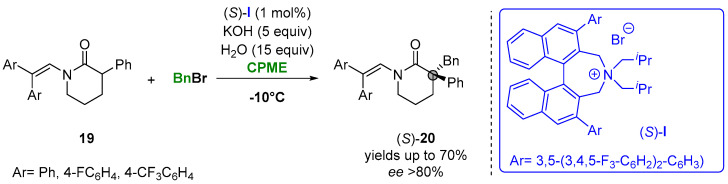
Asymmetric α-benzylation of six-membered lactams in CPME.

In this context, the catalytic asymmetric alkylation of enolates was presented as one of the possible approaches for using biobased solvents. Thus, Maruoka and coworkers reported the asymmetric alkylation of 3-arylpiperidin-2-ones (**19**) under phase-transfer conditions in CPME to access a variety of δ-lactams (**20**) having a chiral quaternary carbon center at the α-position. The reaction of *N*-protected-3-phenylpiperidin-2-ones with alkyl and aryl bromides with the presence of the chiral quaternary ammonium salt (*S*)-**I** achieved the formation of the desired products in a high yield and enantioselectivity in short reaction time (Figure 13). The utility of the procedure was further demonstrated for the synthesis of osanetant, a neurokinin-3 antagonist [92].

**Figure 14:**
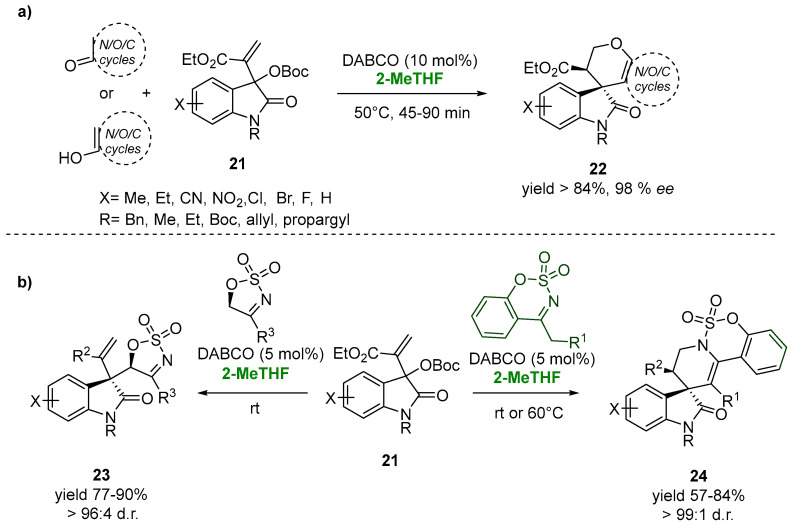
3,3-disubstituted oxindoles via MBH-chemistry in 2-MeTHF (**a**,**b**).

The asymmetric allylic alkylation of the easily accessible Morita–Baylis–Hillman (MBH) carbonates of isatins (**21**) with a variety of enolizable cyclic carbonyl compounds catalyzed by the nucleophilic Lewis base 1,4-diazabicyclo [2.2.2]octane (DABCO, 10.0 mol%) leads to the synthesis of a novel class of spirooxindole-fused-dihydropyran scaffolds (**22**) in excellent yields and optimal diastereoselectivity, up to 99:1 (Figure 14a). The use of 2-MeTHF, an environmentally benign solvent, was chosen as the valuable and ecofriendly choice, ensuring the diasteroselectivity of the process [93].

This biobased solvent also proved its versatility in the case of the reaction of cyclic sulfamidate imines with MBH carbonates of isatins in the presence of a catalytic amount of DABCO for the synthesis of medicinally promising polycyclic spirooxindoles (**23**). The allylic substitution reaction of the MBH carbonates with five-membered cyclic sulfamidate imines at room temperature gave the desired products (**23**), showing, in 2-MeTHF, an excellent yield (87%) and diastereomeric ratio (96:4). In the case of the stereoselective synthesis of the 3,3-tetrahydropyridinyl spirooxindole scaffold (**24**), an increasing of the temperature to 60 °C appeared to be essential for the allylic alkylation-intramolecular aza Michael reaction sequence, also showing the versatility of the green solvent with respect to the variation of temperatures. After 6 h, the reactions resulted in 76–84% yields and excellent *dr* (99:1) (Figure 14b) [94].

**Figure 15:**
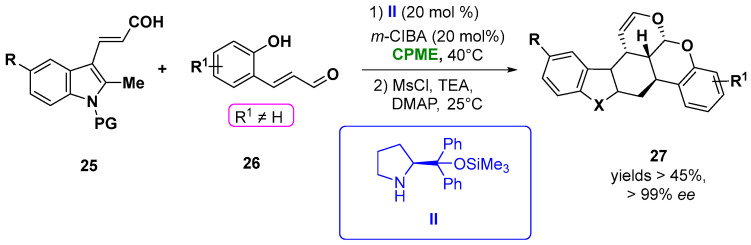
Synthesis of chiral carbazole derivatives from unsaturated indolyl-aldehydes and enals.

CPME was efficiently employed in the construction of chiral polycyclic tetrahydrocarbazole (**27**) and chromane derivatives via an aminocatalytic enantioselective [4 + 2] Diels−Alder of β-indolyl α, β-unsaturated aldehydes (**25**) and α, β-unsaturated aldehydes (**26**) simultaneously activated by the aminocatalyst **II** in the presence of *m*-ClC_6_H_4_CO_2_H (*m*-ClBA), giving the best results in term of the yield and excellent stereoselectivities (in all cases, the *ee* was higher than 99%, d.r. >20:1) (Figure 15). Notably, also in the synthesis of the polycyclic chroman derivatives bearing four chiral centers, a remarkable stereoselectivity was obtained, carrying out the reaction in the same solvent [95].

**Figure 16:**
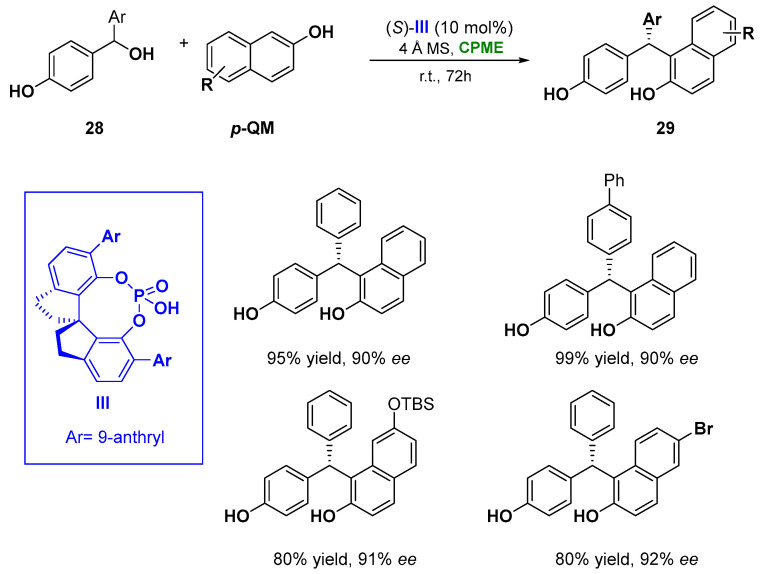
Asymmetric CPA catalyzed of 1,2-diaryls from naphthols and benzylic alcohols.

Notably, in the asymmetric chiral phosphoric acid (CPA) catalyzed 1–6 addition of naphthols (**28**) to a set of *para*-quinone methides (***p*-QMs**), prepared in situ from secondary *p*-hydroxybenzyl alcohols (**6**), CPME proved to be an effective green solution, replacing CHCl_3_ [96] and showing also a superiority in enantioselectivity. The methodology leads to the formation of tertiary stereocenters (**29**), overcoming the limitation of the use of stabilizing bulky substituents (e.g., *t*-Bu) at the α positions of the presynthesized *p*-**QMs** and the restriction in the construction of quaternary stereocenters from tertiary alcohols. The reaction catalyzed by the spirocyclic bis(indane)-derived chiral phosphoric acid **III**, and the use of 4 Å molecular sieves, which facilitate the generation of the *p*-QM intermediate removing the water generated during the process, gave access, at room temperature in 72 h, to a wide range of triarylmethanes in high yields and with excellent enantioselectivity (93% yield, 90% *ee*) (Figure 16) [97]. It is worth underlining that the phosphoric acid **III** serves as a bifunctional catalyst, activating both of the reaction partners via hydrogen bonds.

Due to the major chemical stability of the α-CH bond of CPME, in comparison to other ethereal solvents, such as diisopropyl ether or THF, together with its relatively high boiling point (106 °C), CPME is a suitable alternative in radical reactions. In recent years, a variety of radical addition and reduction methodologies were reported with the use of this solvent, and in the case of photoredox asymmetric catalysis, it also appears to be a valid choice [98].

**Figure 17:**
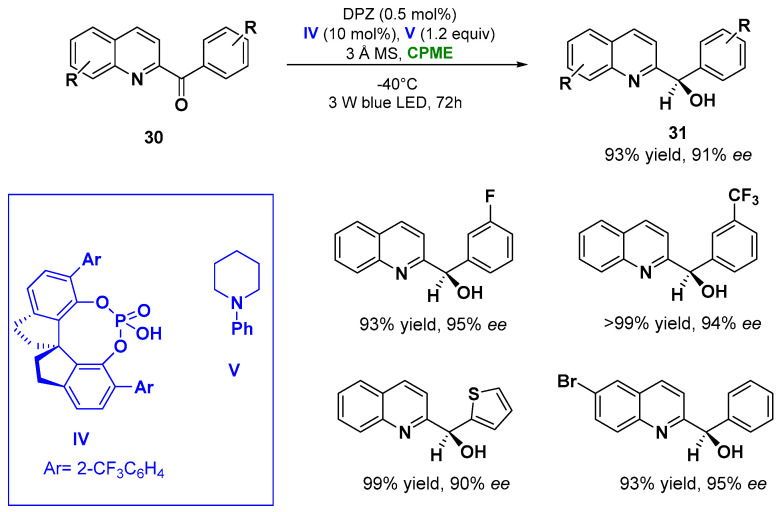
Photocatalytic enantioselective reduction of azaarene-based ketones in CPME employing a chiral phosphoric acid (**IV**) and *N*-phenylpiperidine (**V**).

In point of fact, the enantioselective reduction of azaarene-based ketones (**30**) via visible light performed in CPME at −40 °C using 0.5 mol% of metal-free photosensitizer DPZ, 10 mol% 1,10-spirobiindane-7,70-diol (SPINOL)-based chiral phosphoric acid (CPA) catalyst **IV** and *N*-phenylpiperidine **V** (1.2 equiv. as additive) with the presence of 3 Å MS, furnished the corresponding reduced chiral alcohols (**31**) in 72 h with yields up to 93% and enantiomeric excesses (*ee*) higher than 91%, as indicated in Figure 17 [99].

**Figure 18:**
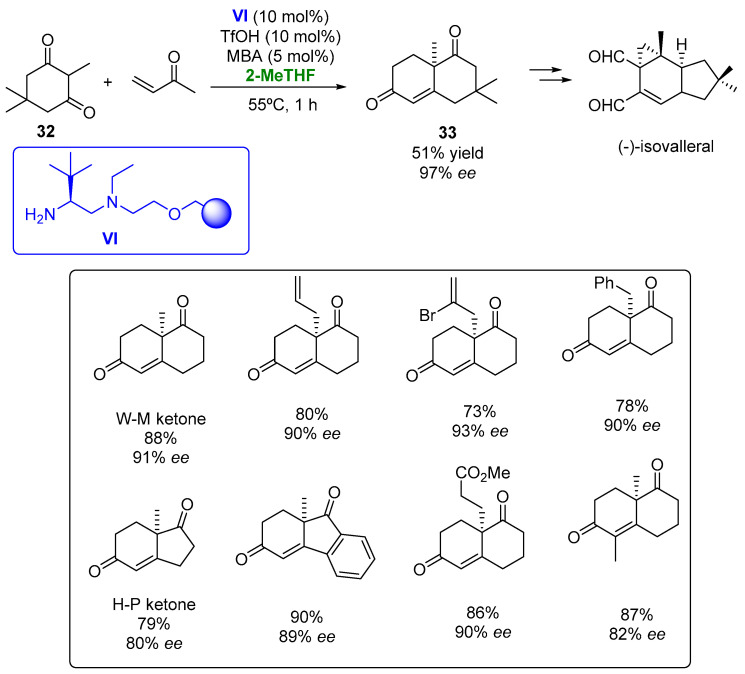
Heterogeneous organocatalyzed preparation of bicyclic diketones employing 2-MeTHF as biobased solvent.

The polystyrene-supported diamine **VI**, derived from L-*tert*-leucine, has been employed as a catalyst in the Robinson annulation to obtain the Wieland–Miescher (W-M) and the Hajos–Parrish (H-P) ketones (Figure 18) [100].

The heterogeneous catalysts were tested in the formation of the W-M ketone, starting from a triketone, with a complete conversion and 92% *ee* in the THF at room temperature being observed when using 10% mol triflic acid and 5 mol% *m*-nitrobenzoic acid (MBA) as the additive. When the reaction was performed in 2-MeTHF, a very similar result was achieved, so this greener alternative was established for carrying out these processes. When the reaction was carried out at 55 °C, a complete conversion was obtained after 1 h with an excellent optical purity (91% *ee*). The optimized conditions were employed for the formation of the W-M ketone, starting from diketone **32** and methyl vinyl ketone, achieving the desired compound after 1 h also with a complete conversion and high enantioselectivity. The substrate scope of this reaction was explored. Bicyclic ketones presenting a [4.4.0] structure and bearing different substituents at the 8a carbon atom were obtained in high optical purities (93% *ee*) after 1–2 h. The H-P ketone and some of its derivatives were also prepared by this methodology with excellent results. The enantioselective preparation of diketone **33**, a valuable intermediate in the synthesis of furanether B or (+)-isovelleral was accomplished in the presence of catalyst **VI** and 2-MeTHF with a 51% yield and 97% *ee*, thus improving the optical purity of the previous methodologies described for the preparation of this compound. The robustness of the organocatalytic method was tested by recycling the catalyst in the preparation of the W-M ketone. High yields and optical purities were achieved after 10 cycles just by increasing the reaction times by 15 min from one reaction to the following one, thus indicating the robustness of the catalytic system.

**Figure 19:**
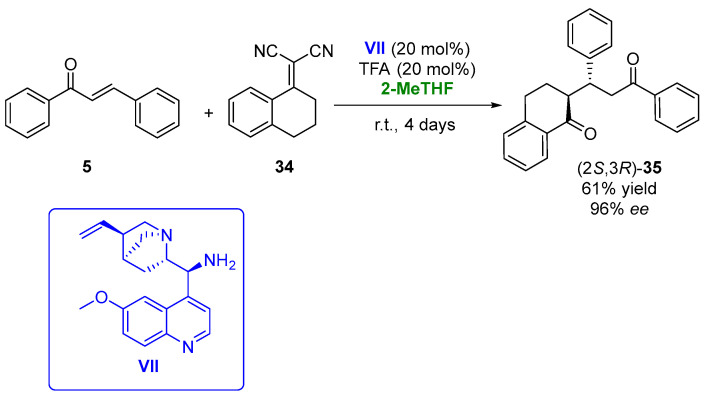
Addition of α,α-dicyanoolefins to chalcones catalyzed by cinchona-based catalysts in 2-MeTHF.

In 2019, the 1,4-addition of α,α-dicyanoolefins (**34**) to chalcones (**5**), employing a bifunctional cinchona-derived organocatalyst (**VII**) in the presence of 2-MeTHF, was described [101]. The initial experiments were carried out in the reaction between the chalcone and α,α-dicyanoolefin in the presence of 9-amino-9-deoxyepiquinine (Figure 19, **VII**, 20 mol%) as the catalyst in THF, using trifle oroacetic acid (TFA, 20 mol%) as the cocatalyst. The chiral adduct (**35**) was recovered with a 56% yield and 97% *ee* after 4 days at room temperature. An evaluation of the reaction solvent led to the best results in the presence of 2-MeTHF, using both 15 or 20% *v*/*v* of the catalyst and cocatalyst, being possible to obtain the final adduct in 61% yield and 96% *ee* after 4 days. Once the reaction solvent was optimized, the procedure was extended to other substituted chalcones and α,α-dicyanoolefins, leading to a family of compounds with antiplasmodial properties in yields around 10–70% and optical purities between 71 and 98%.

**Figure 20:**
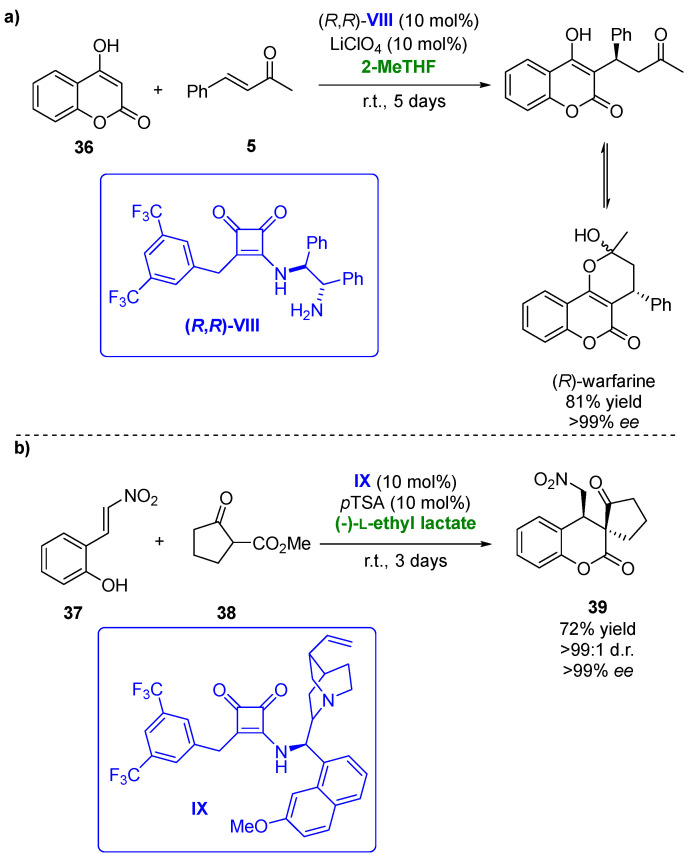
(**a**) Organocatalyzed synthesis of (*R*)-warfarin in presence of biobased solvents. (**b**) Michael addition and cyclation of 2-(2-nitrovinyl)phenols with β-ketoesters catalyzed by squaramide **IX** in biobased solvents.

Biobased solvents have been also applied in asymmetric hydrogen-bonding organocatalytic procedures. In the last few years, chiral squaramides have demonstrated their potential as this type of catalyst, being employed in several transformations with high regio- and/or enantioselectivities [102]. Thus, in 2018, the Michael/hemiketalization reaction of 4-hydroxycoumarines (**36**) with different types of enones (**5**) was carried out using squaramides as organocatalysts [103]. One of the processes analyzed was the addition of 4-hydroxycoumarin to benzylidenacetone yielding (*R*)-warfarin, a valuable anticoagulant (Figure 20a). After 5 days reaction at room temperature, 86% of the desired compound with 90% *ee* was recovered when employing a 1.4-dixoane/MeCN mixture and squaramide (*R*,*R*)-**VIII** (10 mol%) in the presence of 10 mol % of LiClO_4_ as the additive. Once the best catalyst was chosen, a solvent screening was performed, thus analyzing a set of deep eutectic solvents, ionic liquids and biobased solvents, including (-)-L-ethyl lactate, ethylene glycol and its dimethyl ether (monoglyme), PEG 600, CPME and 2-MeTHF. The use of PEG 600 afforded (*R*)-warfarin with an excellent optical purity (97% *ee*) but a modest yield, whereas the best results were obtained in 2-MeTHF (81% yield of enantiopure compound after 5 days). When squaramide (*S*,*S*)-**VIII** was employed as the catalyst, (*S*)-warfarine was obtained with good yield (84%) and a high optical purity (92% *ee*). The substrate scope of the Michael addition in 2-MeTHF was extended to several 4-hydroxycoumarins and benzylidenacetones. The use of different electronic groups in the hydroxycoumarin structure did not affect the optical purities of the final products, whereas this effect was observed for the benzylidenacetones. Those presenting electron-withdrawing groups led to high enantioselectivities, whereas those with electron-donating ones afforded the chiral adducts with low enantioselectivities (52% *ee*).

Recently, a set of bifunctional squaramides have been tested as catalysts in the asymmetric sequential Michael addition and cyclization of a set of 2-(2-nitrovinyl)phenols [104]. These compounds reacted with cyclohexanone, yielding a lactol, which, after a reductive etherification, afforded hexahydro-1*H*-xanthenes. After catalyst screening, the best yields and optical purities were obtained when employing squaramide **IX** in the presence of (*S*)-proline as the cocatalyst and sodium acetate as the basic additive. Compound x was recovered with 83% yield, excellent diastereomeric purity (99:1) and 86% *ee* after 20 days when using DCM as the solvent. The Michael addition step of this sequential procedure was then tested in the presence of biobased solvents, including CPME, 2-MeTHF, (-)-L-ethyl lactate or ethylene glycol. The reactions were faster (8 days reaction time) than in DCM, but for all the solvents tested, lower yields and selectivities were achieved. The best choice for this process was ethylene glycol in the presence of (*S*)-proline and 4-nitrobenzoic acid as the additive, recovering the desired compound in a 61% yield with complete diastereoselectivity and 74% *ee*. The reaction between 2-(2-nitrovinyl)phenol (**37**) and cyclopentanone-2-carboxylate (**38**), a more reactive Michael donor, was also studied. When the process was carried out in DCM, employing **IX** in the absence of a cocatalyst and additive, final product **39** was obtained in a 66% yield with complete diastereoselectivity and 98% *ee* after 1 day (Figure 20b). The process was also analyzed in the presence of different biobased solvents. In this reaction, the use of (-)-L-ethyl lactate allowed to improve the catalytic procedure, as after 3 days, the enantiopure final compound was obtained with a 72% yield, thus demonstrating the advantages of employing this solvent.

## 5. Biocatalytic Approaches Employing Biobased Solvents

The joint use of biocatalysts and biobased green solvents clearly introduces an extra surplus in the sustainability of catalyzed processes. Therefore, several documents can be found in the modern literature covering this “virtuous cycle” synergic effect [29,105], especially for 2-MeTHF [32,33,106] and CPME [38]. In this manuscript, we will focus only on the cases reported in the last five years, covering biocatalyzed procedures developed in green solvents leading to the generation of asymmetry. All the examples described herein were performed employing hydrolases or oxidoreductases, so each type of biocatalyst will be analyzed.

### 5.1. Hydrolases in Presence of Biobased Solvents

**Figure 21:**
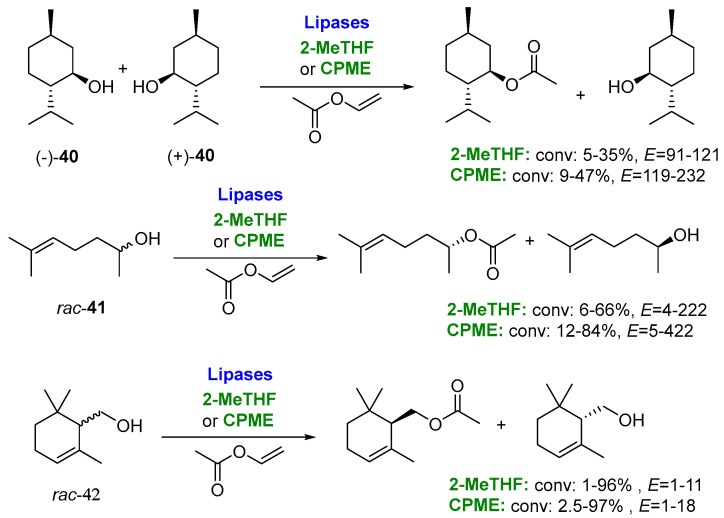
Kinetic resolution of racemic alcohols via lipase-catalyzed acylation with vinyl acetate in 2-MeTHF and CPME.

Regarding 2-MeTHF, the influential work conducted by Simeó et al. [107], reporting the regioselective acylation of several nucleosides catalyzed by lipase B from *Candida antarctica* in 2-MeTHF, can be considered as the real starting point for the modern use of this alternative biobased solvent for biocatalyzed procedures. As commented before, several reviews can be found in the literature, illustrating different applications of this valuable biobased solvent. Undoubtedly, the most frequently reported application of 2-MeTHF in biotransformations is lipase-catalyzed kinetic resolution of alcohols, as these enzymes are highly stable and selective both in aqueous media and in organic solvents [108,109,110]. In this sense, Secundo and coworkers [111] have assessed the utility of 2-MeTHF and CPME in the transesterification of racemic and menthol (**40**), sulcatol (**41**) and α-cyclogeraniol (**42**) with vinyl acetate, catalyzed by several commercial lipases (Figure 21). The activity of lipases in CPME was very similar to that observed in other classical organic solvents (toluene and MTBE) and slightly lower in 2-MeTHF. Interestingly, the enantioselectivity (*E*) [112] was higher in the ecofriendly solvents, although the nature of the substrates influenced the resulting enantioselectivity. Thus, lipase AK (lipase from *Pseudomonas fluorescens*) was found to be the best catalyst for the resolution of racemic **40** in terms of the enantioselectivity, and CPME allowed a faster reaction rate. On the other hand, Novozym 435 (a commercial preparation of the immobilized CALB, lipase B from *Candida antarctica*) was the best choice for the resolution of *rac*-**41**, both in 2-MeTHF and CPME, leading to an excellent enantioselectivity and reaction rate. For the primary alcohol *rac*-α-**42**, all the tested lipases showed a low enantioselectivity. Remarkably, the way of preparing the biocatalyst showed an influence on the lipase behavior; in fact, through lyophilization after dissolving it at pH 8.0, lipase AK showed a higher activity in the acetylation of (±)-**40** in CPME, even increasing this effect by lyophilizing in the presence of additives, such as MeOPEG or sucrose.

**Figure 22:**
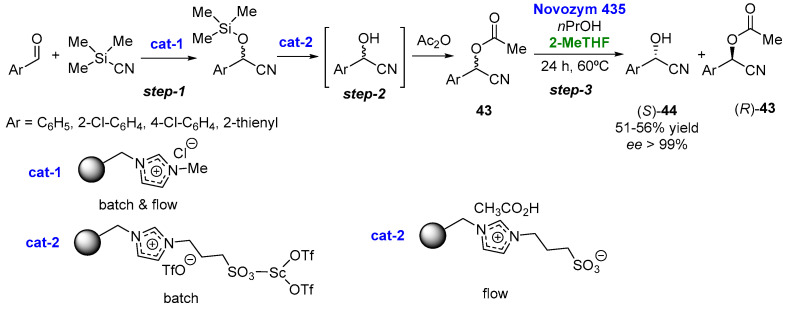
Chemoenzymatic cascade leading to pure chiral cyanohydrins.

In another example, Peris et al. [113] have reported the arrangement of various consecutive multicatalytic steps by combining organocatalytic-supported ionic-liquid-like phases (SILLPs) with Novozym 435 for the preparation of optically pure chiral cyanohydrins **44** (Figure 22) through a kinetic resolution of the racemic acetylated cyanohydrins **43** via transesterification with propanol, leading in all cases to excellent yields and optical purity.

**Figure 23:**
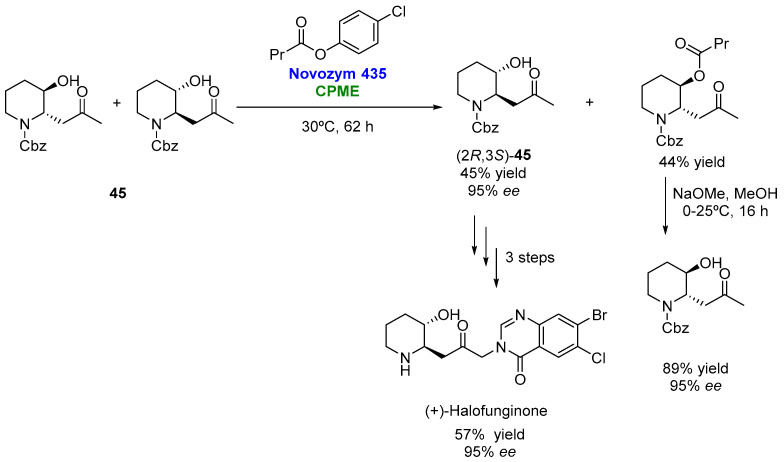
Chemoenzymatic synthesis of (+)-halofunginone employing CPME for the biocatalyzed kinetic resolution.

In a very recent publication, de Marchi et al. [114] have reported the synthesis of both enantiomers of a key building block for the synthesis of halofuginone (Figure 23), a molecule possessing antiprotozoal activity against several strains of *Eimeria* in poultry, as well as some other pharmacological activities. Hence, racemic *trans* benzyl-3-hydroxy-2-(2-oxopropyl)piperidine-1-carboxylate (**45**) was effectively resolved via transesterification. Of all the acyl donors tested, *p*-chlorophenylbutyrate (PCPB) was the best option, while CPME (not previously dried) led to the best conversion and reaction rates. The results with 2-MeTHF in the previous screening with vinyl butyrate as the acyl donor were slightly worse, so the authors used CPME for the synthetic procedure.

**Figure 24:**
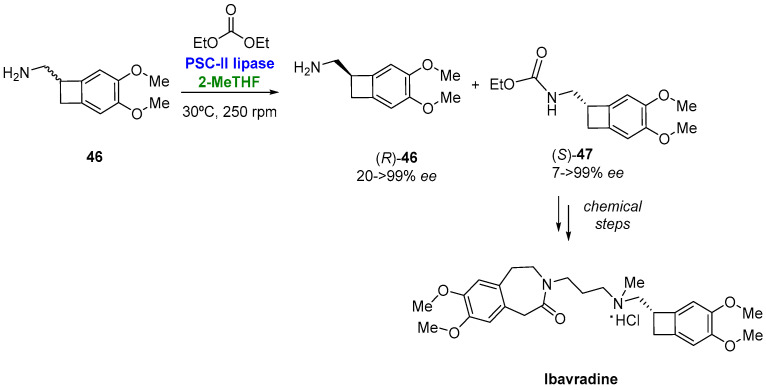
Chemoenzymatic preparation of optically pure Ivabradine.

Chiral amines can also be resolved by a lipase-catalyzed resolution. Thus, Pedragosa-Moreau et al. [115] reported the use of the lipase from *Pseudomonas cepacia* (PSC-II) for the kinetic resolution of racemic (3,4-dimethoxybicyclo[4.2.0]octa-1,3,5-trien-7-yl)methanamine (**46**) via alkoxycarbonylation with diethyl carbonate in 2-MeTHF. In this way, depicted in Figure 24, the resulting (*S*)-carbamate (**47**) was used as the chiral building block for the preparation of Ivabradine (the treatment of stable angina pectoris in cases of intolerance or contraindications for β-blockers), which could be isolated in a 30% overall yield. For the kinetic resolutions, these authors also tested CPME, which was also a good option, although both conversion and enantioselectivity were slightly lower.

The use of a CO_2_-expanded liquid (CXLs) is becoming increasingly used [116]. These CXLs are organic liquids into which a large amount of CO_2_ has been dissolved under pressure, so that the CO_2_ content modifies and tunes many of the liquid properties. In this sense, the use of CO_2_-expanded phases with 2-MeTHF has been reported, leading to sustainable solvents with tailored properties [117,118,119]. Thus, by expanding the carbon dioxide (up to 10 bar) in 2-MeTHF, it is possible to alter the solvent properties, such as polarity or hydrophobicity; in biocatalysis, CO_2_-expanded 2-MeTHF can be used for improving kinetic resolutions of bulky substrates, usually showing low or no activity in 2-MeTHF.

**Figure 25:**
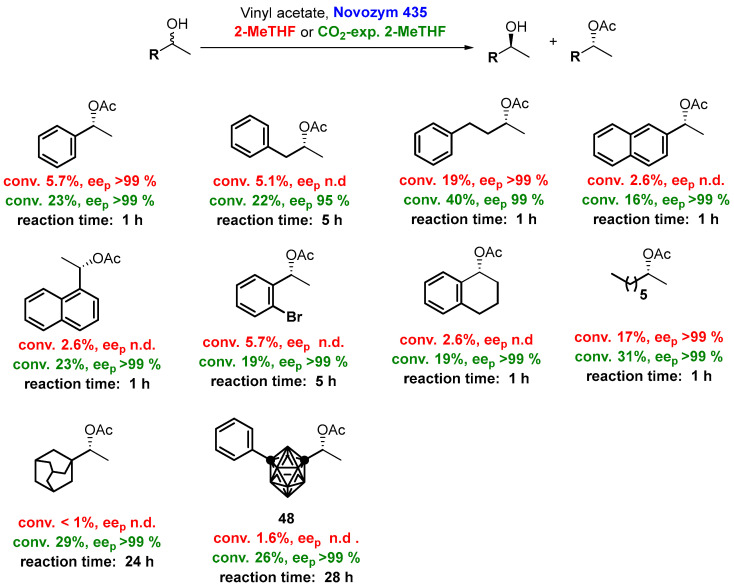
Lipase-catalyzed resolution of bulky alcohols using CO_2_-expanded 2-MeTHF.

Thus, Hoang et al. [117,118] have tested this binary solvent in the resolution of several bulky secondary alcohols via lipase-catalyzed transesterification, showing an excellent enantioselectivity. Apparently, some flexibilization of the protein structure occurs in the expanded solvent, thus enabling the acceptance of extremely bulky substrates. As can be seen from the data in Figure 25, the use of CO_2_-expanded 2-MeTHF increases the conversion compared to those obtained using only a biosolvent, not affecting the enzymatic enantioselectivity. Furthermore, these reactions can be scaled-up, as shown by Hoang et al. [118] for the lipase-catalyzed kinetic resolution of *rac*-1-adamantylethanol, subsequently performed in a grams (2.13 g, 11.8 mol), allowing the separation of the corresponding (*R*)-acetate (1.21 g, 46% yield, *ee* > 99%) and (*S*)-alcohol (0.94 g, yield 44%, *ee* > 99%). Noteworthy, this kinetic resolution would be impossible in only sole liquid CO_2_ because of the very low solubility of the substrate. Another very attractive substrate is 1-(7-phenyl-1,7-dicarba-closo-dodecaboran-1-yl)ethanol (**48**), bearing an icosahedral boron cluster (*m*-carborane, C_2_B_10_H_12_) with a bulky spherical surface, for which the reaction resulted in a very high conversion in the CO_2_-expanded 2-MeTHF but a poor conversion observed in the neat biosolvent.

**Figure 26:**
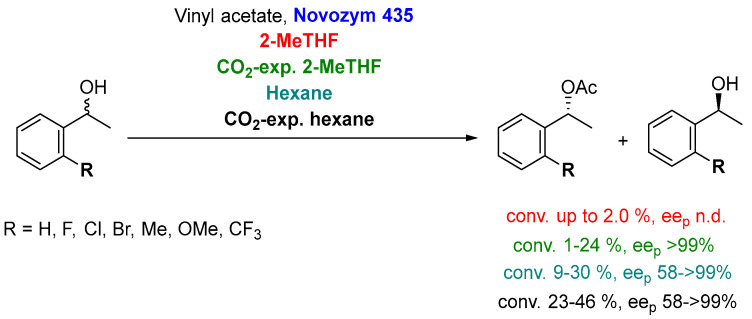
Lipase-catalyzed resolution of several *ortho*-substituted 1-phenylethanols in different solvents.

In order to explain the high conversion observed for CALB in the kinetic resolution of bulky molecules, Hoang et al. [117] suggested three possibilities: (a) the formation of carbamates from CO_2_ and the free amine groups (such as lysine) on the surface of the lipase, leading to beneficial conformational changes, (b) an increase in the enzyme flexibility and/or less compactness of CALB when soaked in CO_2_ media, allowing an increased recognition of bulky substrates, and/or (c) the enhanced transport and physicochemical properties of the expanded 2-MeTHF compared to the neat biosolvent. Another paper from same research group has shown that, although CO_2_-expanded 2-MeTHF can be used for the CALB-catalyzed kinetic resolution of several *ortho*-substituted 1-phenylethanols, better results are observed employing CO_2_-expanded hexane [119], as shown in Figure 26.

**Figure 27:**
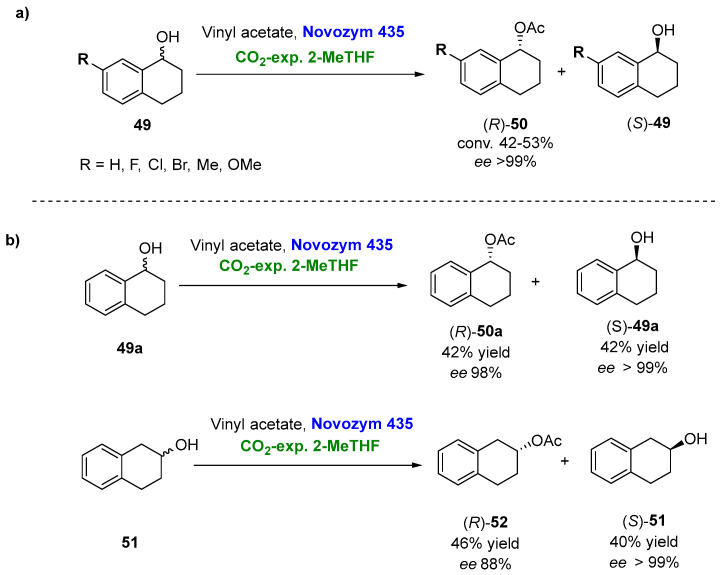
(**a**) Lipase-catalyzed resolution of racemic tetralols using CO_2_-expanded 2-MeTHF, analytical scale. (**b**) Scaled (20 times) kinetic resolution of racemic 2-tetralol (**51**).

Very recently, Suzuki et al. [120] have extended the applicability of CO_2_-expanded solvents not only to 2-MeTHF and hexane but also to some other biobased solvents, such as γ-valerolactone, diethyl carbonate, (+)-limonene, (-)-limonene and *p*-cymene, in the kinetic resolution of racemic-substituted 1-tetralols, as depicted in Figure 27a.

Using neat solvents, the highest conversions were reported when using petrol-based solvents (*n*-hexane followed by *i*-Pr_2_O, max. conversion 28%) for both 1-tetralol (**49a**) and 2-tetralol (**51**). Authors suggested that the lower conversions obtained with biobased solvents (e.g., 2-MeTHF below 10%) were caused by their lower hydrophobicity. Once again, testing the performance of CO_2_-expanded liquids (concentration of biobased liquid or petroleum-derived liquids 10% *v*/*v*, 10 mL, 6.0 MPa), the conversions and reaction rates dramatically improved for all the biobased or petroleum-derived liquids, CO_2_-expanded 2-MeTHF being the best option (conversion up to 43%). The reaction was scaled by 20 times for 1- and 2-tetralol (Figure 27b), allowing the isolation of the corresponding (*R*)-acetates and (S)-alcohols with excellent enantioselectivities (up to *ee* > 99%).

Another very attractive strategy for the efficient lipase-catalyzed transesterification of largely hydrophobic compounds is the use of Pickering Emulsions (PEs), nanoparticle-stabilized emulsions consisting of enzymes immobilized in water droplets stabilized by nanoparticles and surrounded by solvent molecules containing the substrates [121]. These systems can be easily applied when working in a continuous mode. Then, the transesterification of 1-phenylethanol with vinyl butyrate catalyzed by *Candida antarctica* lipase A (CalA) was described using CPME [122,123], with space-time yields around 120 mg L^−1^ h^−1^, although the author did not report the enantioselectivity. A later paper from Heyse et al. [124] expanded the study to other lipases in the same system, but once again, no enantioselectivity was reported.

**Figure 28:**
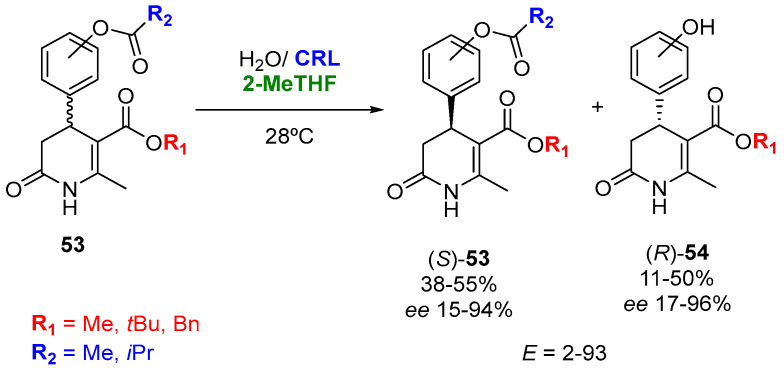
Lipase-catalyzed preparation of several 3,4-DHP-2-ones in water/2-MeTHF.

Biosolvents can also be used as the cosolvent for the lipase-catalyzed kinetic resolution of esters in aqueous media. For instance, Torres et al. [125] reported the synthesis of optically active 4-(3-acetoxyphenyl)-5-(alkoxycarbonyl)-6-methyl-3,4-dihydropyridin-2-ones (3,4-DHP-2-ones, **53**), components of natural products [126,127], displaying many therapeutic effects [128,129,130]), by means of a lipase-catalyzed hydrolysis reaction to yield alcohols **54** using 2-MeTHF as the cosolvent, as shown in Figure 28. The best results for the enzymatic hydrolysis of the phenolic esters in 2-MeTHF/water (99/1 *v*/*v*) were obtained using lipase from *Candida rugosa* (CRL), with a good optical purity (enantiomeric excesses between 94 and 99%) and yields.

### 5.2. Biobased Solvents in Reactions Catalyzed by Oxidoreductases

**Figure 29:**
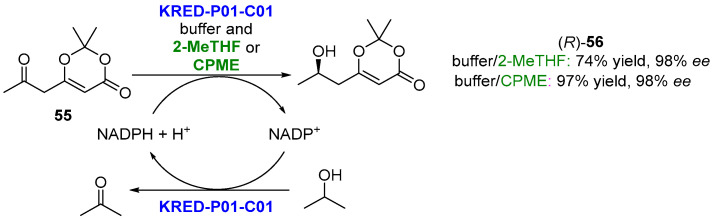
KRED-catalyzed reduction of β-ketodioxinones (**55**) in biphasic media.

Bio-derived solvents can be also used in biotransformations catalyzed by redox enzymes, acting as cosolvents. A recent review by Aranda and de Gonzalo [131] illustrated this research field. Ketoreductases (KREDs), enzymes which catalyze the reversible NAD(P)H-dependent transformation of carbonyl compounds into the corresponding alcohols, have been described to perform well in water/biosolvent biphasic systems. As an example, the enantioselective reduction of β-ketodioxinones (**55**) by means of ketoreductases to afford β-hydroxydioxinones, useful building blocks to produce a variety of natural products, was reported by Betori et al. [132] (Figure 29).

The use of a commercially engineered ketoreductase (KRED-P01-C01 from Codexis) led to the best results in buffers containing NADPH and *iso*-propanol for the nicotinamide cofactor regeneration. When the bioreductions were developed in the presence of ethereal solvents, such as 2MeTHF or CPME, at 10% *v*/*v* concentration, the reaction yields were increased: 97% yield and 98% *ee* using CPME and 74% yield and 98% *ee* for 2-MeTHF. These optimized conditions were extended to the bioreduction of other β-ketodioxinones, obtaining the final products with excellent yields (>90%) and selectivities (>90% *ee*) for almost all the substrates [132]. It was also possible to scale up the bioreduction using a substrate concentration of 100 g/L; after 72 h, 20 g of the (*R*)-**56** was isolated with 99% yield and >98% *ee* after a simple extraction. Finally, it was also possible to obtain the (*S*)-**56** at 97% conversion, although with a smaller optical purity (*ee* 79%), conducting the reaction in similar conditions but employing a ketoreductase with opposite enantiopreference.

**Figure 30:**
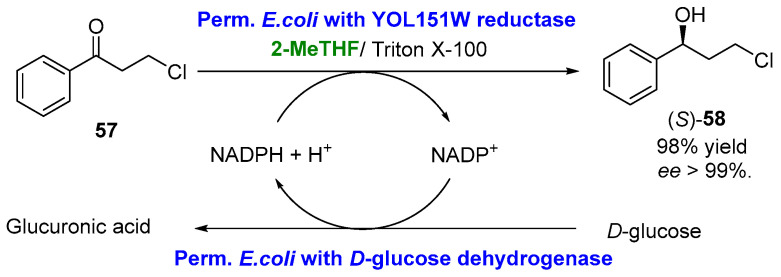
Whole cells-catalyzed reduction of a chloroketone using 2-MeTHF as cosolvent.

Redox biotransformations can also be developed using whole cells instead of pure free enzymes. In these systems, the crucial cofactor regeneration becomes easier as the metabolic machinery of the cell oversees it [133]. Following this methodology, biosolvents can also be used. For instance, Tian et al. [134] reported the use of permeabilized whole-cells in the stereoselective bioreduction of the prochiral ketone 3-chloro-1-phenyl-1-propanone (**57**), using 2-MeTHF as the cosolvent (Figure 30). The reaction product, (*S*)-3-chloro-1-phenylpropanol (*S*)-**58**, is a building block to furnish antidepressant drugs, such as fluoxetine, tomoxetine or nisoxetine. For the bioreduction, permeabilized recombinant cells of *Escherichia coli* containing the YOL151W reductase from *Saccharomyces cerevisiae* were used, while permeabilized recombinant cells of *E. coli* containing *D*-glucose dehydrogenase was the option for NADPH regeneration. The use of 2-MeTHF as the cosolvent (1–7% *v*/*v*), together with a surfactant (Triton X-100), was crucial to increase the low water solubility of the substrate up to 60 mM, leading to 98% yields and an ***ee*** > 99%.

**Figure 31:**
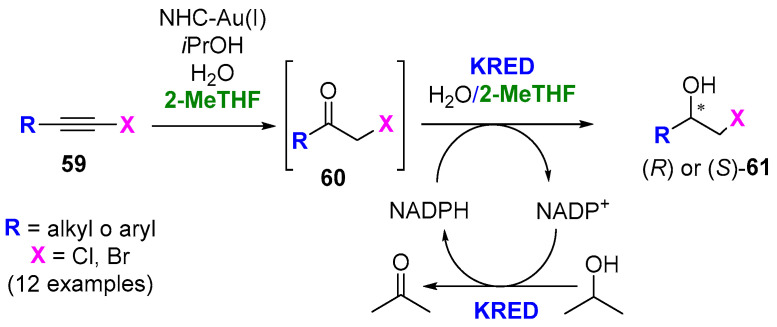
Chemoenzymatic cascade for producing enantiopure halohydrin from haloalkynes in aqueous/2-MeTHF medium.

Recently, the group of Lavandera and Gotor-Fernández have reported a concurrent chemoenzymatic cascade to convert different haloalkynes (**59**) into enantiopure halohydrins (**61**) in an aqueous/2-MeTHF medium by combining a gold(I) *N*-heterocyclic carbene (NHC) and stereo-complementary KREDs, as shown in Figure 31 [135].

Hence, several alkyl- or aryl-substituted haloalkynes were converted into the corresponding chloro- or bromohydrins in good-to-high yields (65−86%). The standard substrate for optimization was (chloroethynyl)benzene (R = Ph, X = Cl), and the best results were obtained using [1,3-bis(2,6-diisopropylphenyl)-imidazol-2-ylidene] [bis(trifluoromethanesulfonyl)imide]gold-(I) (IPrAuNTf_2_) as the catalyst, two equivalents of *iso*-propanol and 2-MeTHF (10 or 20%), leading to conversions up to 99% (up to 94% yield after column chromatography) at 4 °C after 16 h. For the bioreduction of ketones **60**, after testing twenty-three KREDs, the author found that commercially available KRED-P1-A04, KRED-P1-A12 and KRED-P2-H07 (from Codexis) and lyophilized cells of *E. coli* overexpressing ADH from *Lactobacillus brevis* (*Lb*ADH) were adequate for the preparation of (*S*)-halohydrins in quantitative conversion. On the other hand, only the ADH-A (alcohol dehydrogenase from *Rhodococcus ruber*) was useful for preparing (*R*)-halohydrin in 98% conversion. Finally, the concurrent cascade was performed using again (chloroethynyl)benzene, IPrAuNTf_2_ and *Lb*ADH, leading to (*S*)-2-chloro-1-phenylethan-1-ol with a 96% conversion (87% yield after purification) and an *ee* > 99%.

**Figure 32:**
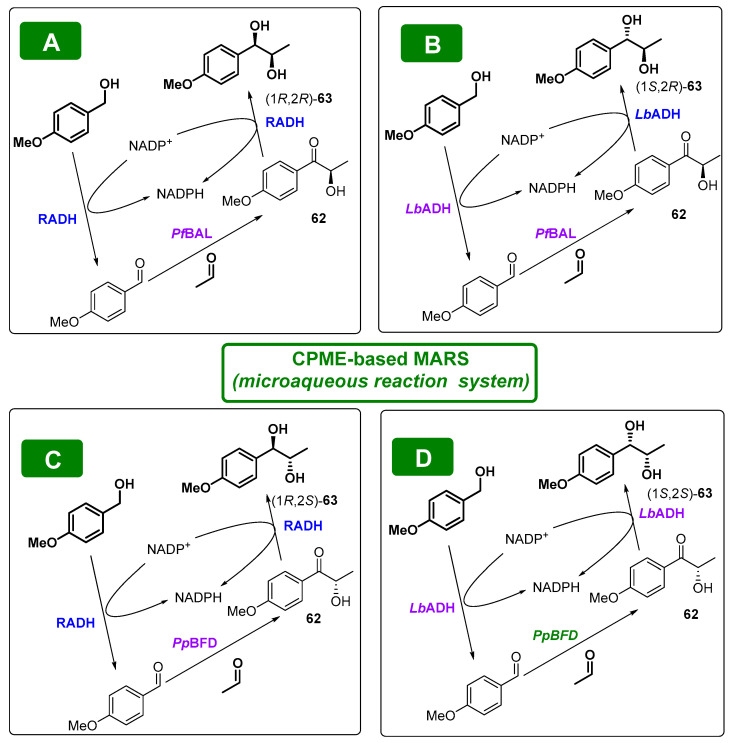
Biocatalytic cascades furnishing the four stereoisomers of **63** in CPME-based MARS system; (**A**) *Pf*BAL and RADH leading to (1*R*,2*R*)-diol; (**B**) *Pf*BAL and *Lb*ADH leading to (1*S*,2*R*)-diol, (**C**) *Pp*BFD and RADH leading to (1*R*,2*S*)-diol and (**D**) *Pp*BFD and *Lb*ADH leading to (1*S*,2*S*)-diol.

In a very elegant strategy, Rother and coworkers [136] have described four stereocomplementary biocatalytic cascades, furnishing the four stereoisomers of 4-methoxyphenyl-1,2-propanediol (**63**) with excellent *ee* and de values of >99%, as indicated in Figure 32.

In this system, two complementary ThDP-dependent carboligases ((*R*)-selective benzaldehyde lyase from *Pseudomonas fluorescens* (*Pf*BAL) or (*S*)-selective benzoylformate decarboxylase from *Pseudomonas putida*) lead to the two enantiomers of a hydroxyketone (**62**) from *p*-methoxybenzaldehyde and acetaldehyde. Subsequently, the intermediate hydroxyketone is reduced using two complementary alcohol dehydrogenases: ADH from *Ralstonia* sp. (RADH, (*R*)-selective) or *Lactobacillus brevis* (*Lb*ADH, (*S*)-selective). One of the pivotal points of the cascades is the use of *p*-methoxybenzyl alcohol as the cosubstrate, which is oxidized to furnish the aldehyde used for the carboligation. This methodology allowed for efficient NADPH cofactor recycling while removing the co-product formed during the ADH-catalyzed step. The other one is the use of a microaqueous reaction system (MARS), where lyophilized (death) whole cells (LWC, much more active in organic conditions) containing high amounts of the recombinant biocatalysts are suspended in a substrate−CPME mixture. Thus, these self-sufficient cascades led to an atom economy of 57% in the sequential mode and 99% in the simultaneous mode, with excellent *ee* and de values of >99% and space-time yields of up to 165 g/L day. Similar cascades but using aqueous media have also been reported by Rother´s group [137,138].

**Figure 33:**
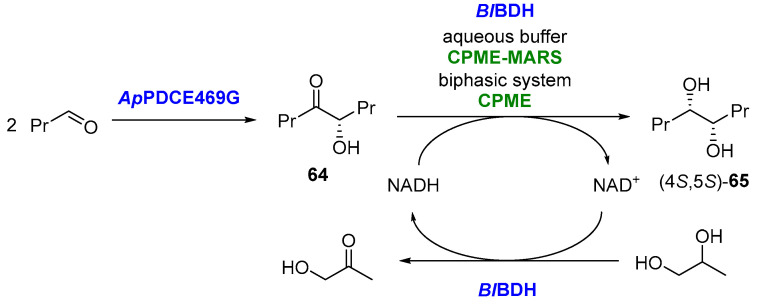
Biocatalytic cascade leading to (4*S*,5*S*)-octane-4,5-diol.

This same group has published another simultaneous one-pot two-step cascade for the preparation of (4*S*,5*S*)-octanediol (**65**), as shown in Figure 33 [139].

In this cascade, initially, a benzoin-type condensation of two butanal molecules catalyzed by *Ap*PDCE469G (a variant of the pyruvate decarboxylase from *Acetobacter pasteurianus*) produced (*S*)-butyroin (**64**), which is subsequently reduced by *Bl*BDH (butanediol dehydrogenase from *Bacillus licheniformis*) to generate (4*S*,5*S*)-**65**. The cofactor regeneration is carried out using 1,2-propanediol as the auxiliary substrate, being oxidized to hydroxyacetone (coproduct). Notably, *Bl*BDH has a high affinity toward the intermediate (*S*)-**64** but not to the starting butanal. Three different operational approaches, namely aqueous monophasic, organic monophasic (CPME-MARS) or biphasic system (also using CPME), were compared, leading in all cases to excellent stereoselectivity (*ee* and *de* > 99%). This last system allowed the best results in terms of the space-time yield (8.6 g/L day) and lowest specific energy demand (249 kJ/g) for product purification, as the biphasic system resulted in ISPR (in situ product removal) because of the hydrophobicity of the product, which goes to the CPME phase, while the catalyst, cosubstrate and coproduct are kept in the aqueous phase.

**Figure 34:**
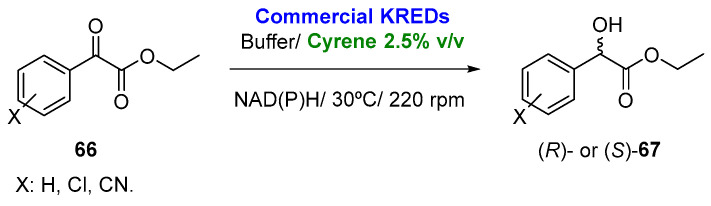
Bioreduction of different α-ketoesters employing KREDs in presence of Cyrene as cosolvent.

Dihydrolevoglucosenone (Cyrene^TM^) has been recently employed in biocatalytic reactions, such as the regioselective esterification of glycerol in the presence of benzoic acid catalyzed by a CAL-B cross-linked preparation, leading to the final esters with excellent conversions when employing Cyrene concentrations of 40% *v*/*v* [140]. Its application in asymmetric biocatalytic procedures was first described in 2021, when Cyrene was applied as the cosolvent in the bioreduction of a set of α-ketoesters (**66**) catalyzed by commercially available KREDs from Codexis [141], as shown in Figure 34. The use of Cyrene at concentrations of 2.5% *v*/*v* led to the corresponding (*S*)- and (*R*)-hydroxyesters (**67**), with complete conversions and enantioselectivities around 90% *ee* when using the biocatalysts KRED P2-D03, KRED P2-D12 or KRED 130. The bioreductions catalyzed by KRED P2-D03 can be performed at Cyrene concentrations up to 30% *v*/*v* with only a small loss on both the activity and the selectivity. α-Ketoesters can be employed at concentrations of 1.0 M in this cosolvent (2.5% *v*/*v*) with a good productivity (144.0 g of enantiopure hydroxyester per liter and day), higher to that achieved in the absence of this cosolvent.

## 6. Summary

The intrinsic (beneficial) characteristics of solvents in determining pivotal aspects of chemical reactions often constituted their Achille’s heel in terms of sustainability. In particular, the adherence to the Anastas and Warner’s Green Chemistry Principles has been rather limited, thus narrowing the significance of transformations of innate synthetic potential: in this sense, the notorious implementing effects on the stereocontrol exerted by the apolarity of the solvents—as well illustrated by classical ethers, halomethanes or hydrocarbons—posed severe issues which nowadays can be conveniently circumvented with the so-called green analogues. These biobased solvents represent a valuable alternative to the classical solvents, in terms of toxicity and biodegradability, being obtained from renewable sources. The selected cases presented in this review indicate the full adaptability of such attractive media to a plethora of conceptually distinct stereochemical logics, thus conjugating the modern needs of synthesis with acceptable environmental metrics. Biobased solvents have been successfully applied in metal-based, organocatalytic and biocatalytic procedures, showing in most cases improved results regarding their “classical” counterparts.

## Figures and Tables

**Table 1:** Some properties of 2-MeTHF, CPME and Cyrene^TM^, usual biobased solvents employed in asymmetric synthesis.

Solvent	Boiling Point (°C)	Freezing Point (°C)	Viscosity (cP)	Water Solubility 20 °C (mg/Kg)	Rat Oral LD_50_ (mg/Kg)	Rabbit Dermal LD_50_ (mg/kg)
2-MeTHF	80	−137	0.473	140,000	2000	2000
CPME	106	−140	0.555	1100	2000	4500
Cyrene^TM^	226			miscible		

## Data Availability

Not applicable.
